# Faraday forcing of high-temperature levitated liquid metal drops for the measurement of surface tension

**DOI:** 10.1038/s41526-018-0044-1

**Published:** 2018-05-14

**Authors:** Nevin Brosius, Kevin Ward, Satoshi Matsumoto, Michael SanSoucie, Ranga Narayanan

**Affiliations:** 1 Department of Chemical Engineering, University of Florida, Gainesville, FL 32611 USA; 2 Human Spaceflight Technology Directorate, Japan Aerospace Exploration Agency, Tsukuba, Ibaraki 305-8505 Japan; 3 NASA Marshall Space Flight Center, Huntsville, AL 35812 USA

## Abstract

In this work, a method for the measurement of surface tension using continuous periodic forcing is presented. To reduce gravitational effects, samples are electrostatically levitated prior to forcing. The method, called Faraday forcing, is particularly well suited for fluids that require high temperature measurements such as liquid metals where conventional surface tension measurement methods are not possible. It offers distinct advantages over the conventional pulse-decay analysis method when the sample viscosity is high or the levitation feedback control system is noisy. In the current method, levitated drops are continuously translated about a mean position at a small, constant forcing amplitude over a range of frequencies. At a particular frequency in this range, the drop suddenly enters a state of resonance, which is confirmed by large executions of prolate/oblate deformations about the mean spherical shape. The arrival at this resonant condition is a signature that the parametric forcing frequency is equal to the drop’s natural frequency, the latter being a known function of surface tension. A description of the experimental procedure is presented. A proof of concept is given using pure Zr and a Ti_39.5_Zr_39.5_Ni_21_ alloy as examples. The results compare favorably with accepted literature values obtained using the pulse-decay method.

## Surface tension: picking up good vibrations

A novel method for measuring the surface tension of droplets of liquid metal is developed by researchers in the USA and Japan. Many of the techniques used to investigate the properties of an interface between liquid and air are not applicable to very high temperature fluids. The method now demonstrated by Nevin Brosius, Kevin Ward, and Ranga Narayanan from the University of Florida and collaborators from NASA and Japanese Aerospace Exploration Agency (JAXA), which they call Faraday forcing, employs a continuous periodic electrostatic force that causes a levitating drop to vibrate. The amplitude of these vibrations reaches a maximum when the driving frequency equals the natural frequency of the drop. This value can then be used to calculate the surface tension. The team demonstrate the viability of their technique with pure zirconium and an alloy of titanium, zirconium, and nickel.

## Introduction

The principal idea behind the current study is to employ a method of surface tension measurement that is particularly suited to high temperature metals and metal alloys. This method, called Faraday forcing due to its similarity to typical Faraday instability oscillatory forcing techniques, marks the first time that *continuous* parametric forcing has been used to induce resonance within levitated droplets for correlation to surface tension. In a typical Faraday instability experiment,^1^ a fluid layer is oscillated about its mean at a fixed amplitude with increasing frequency until the imposed parametric frequency is equal to the system’s natural frequency. At this point, the fluid system enters a state of resonance, and the instability is manifested by vigorous flow, attended by definite interfacial modal structures. In the current study, periodic electrostatic forcing can cause pattern formation on the surface of a levitated fluid sphere when the frequency of the forcing resonates with the sphere’s natural frequency. These patterns, upon excitation, are expressed as oscillating modal structures which deviate from the otherwise spherical shape of the levitated droplet. Drop levitation significantly reduces gravitational effects and allows for containerless experimentation, eliminating sidewall effects that have been shown to be detrimental to the agreement between Faraday instability theory and experiments.^2^ Though Faraday forcing has never been used to measure surface tension in *levitated* samples, similar resonant forcing techniques have been employed in *constrained* geometries for this measurement. For example, Chung et al.^3^ correlated the measurement of interfacial tension to the behavior of capillary wave resonance modes utilizing quasi-elastic laser scattering to measure the resonance. Similar methods have been studied by Wada et al.^4^ and Pigot and Hibara.^5^ Resonant frequencies are determined by Iino et al.^6^ by purposefully exciting standing wave instabilities on an interface within a cylindrical container using electrostatic oscillations. A similar method was later employed by Tsukahara et al.,^7^ who also related the experimentally determined resonant frequency to the surface tension of multiple fluids using expressions for the fluids’ natural frequencies. Non-resonance methods which employ correlation between the appearance of fluid instabilities and surface tension have also been explored.^8–10^

One of the weaknesses in using these types of methods or traditional surface tension measurement methods like pendent drop tensiometers or liquid bridges is their reliance on constrained geometries. This creates significant challenges in using high temperature samples, such as liquid metals. To process these types of samples, advanced techniques such as electrostatic levitation (ESL) have been developed. The use of ESL for containerless processing has allowed for the study of many thermophysical properties for high temperature liquid metals, including density, surface tension, viscosity, and many others.^11^ Of particular interest to the current work is the use of ESL for the measurement of surface tension. Traditional methods for this measurement rely on the use of the equation for the natural frequency of a spherical drop, originally derived by Rayleigh^12^ and also given by Lamb.^13^ Adding a correction to this equation to account for surface charge^14^ results in:

1 $$\omega _n^2 = n(n - 1)(n + 2)\frac{\sigma }{{\rho R_0^3}}\left( {1 - \frac{{Q^2}}{{64{\mathrm {\pi}} ^2R_0^3\sigma \epsilon _0}}} \right),$$

where *ω* is the natural frequency of the drop, *ρ* is its density, *σ* the surface tension, *R*_0_ the drop radius, *n* is the *n*th mode of oscillation of the droplet, *Q* is the drop charge, and $\epsilon _0$ is the permittivity of free space. This equation is derived through inviscid theory, and approximates the natural frequencies for spherical liquid metal drops due to their low kinematic viscosities. For example, the kinematic viscosity of molten Zr at its melting point is 80% of that of water. In the case of liquid metals, the correction term arising due to interfacial charge is typically very small, resulting in changes to the surface tension on the order of 1%. For this reason, the correction term is ignored in the surface tension measurements shown in the present work.

In typical non-constant forcing experiments, i.e., the pulse-decay method, the drop is pulsated by first translating it about its mean position at an assigned frequency. When this assigned frequency is close to the natural frequency, the drop will execute an *n* = 2 mode, at which point the pulsation is ceased. After the imposed pulsation ceases, the amplitude of the oscillatory prolate-oblate deformation of the spherical drop decays with time. Image analysis is used to fit this decay to a sinusoidal, exponentially decaying function with a given frequency, which is then taken to be the natural frequency of the droplet.^15^ This frequency is input into Eq. (1) to deduce the surface tension of the material. Since the feedback system to maintain the drop in its levitated state must re-stabilize the drop after this removal, additional perturbations are unintentionally imposed on the droplet during the initial stages of the decay due to control system noise. These perturbations act to excite all manners of modes within the droplet, which can affect the decay response frequency through non-linear interactions between modes. Thus, the natural frequency obtained from the pulse-decay method is a non-linear composite of the natural frequencies for the various modes. The underlying problem with this method is this unintentional excitation of all other modes upon removing the forcing oscillation.

The Faraday forcing method in this study differs from the conventional pulse-decay technique due to its *continuous* nature. As the forcing is continuous, the oscillation mode remains excited throughout the course of the measurement at resonant conditions, much like the standing waves observed in traditional Faraday instability^16^ experiments. This forcing method is akin to that of ref. ^6^ and ref. ^7^, but is utilized on high temperature samples that have already been electrostatically levitated, thus removing sidewall effects from the problem and resultant model. This allows for the determination of the resonant frequency through imaging the droplet’s departure away from a spherical shape. The resonant frequency is taken to be the frequency of forcing where the drop’s deviation from a sphere into prolate and oblate shapes is at its maximum. When utilizing the Faraday forcing method, no curve fitting is required. Since a decaying function is not fit post-experiment, the continuous nature of the Faraday forcing method inherently avoids feedback system interference which is present in traditional methods. In addition, highly viscous samples present difficulties for the traditional method due to fast damping. These problems could potentially be alleviated by utilizing the Faraday forcing method, upon re-deriving an expression similar to Eq. (1), but where viscosity is now taken into account.^13,17^ Notwithstanding the benefits of continuous forcing, the method presented in this study is unable to yield the viscosity of the sample. Thus, the pulse-decay method would be needed in conjunction with the current method for the determination of both surface tension and viscosity.

## Results

**Fig. 1:**
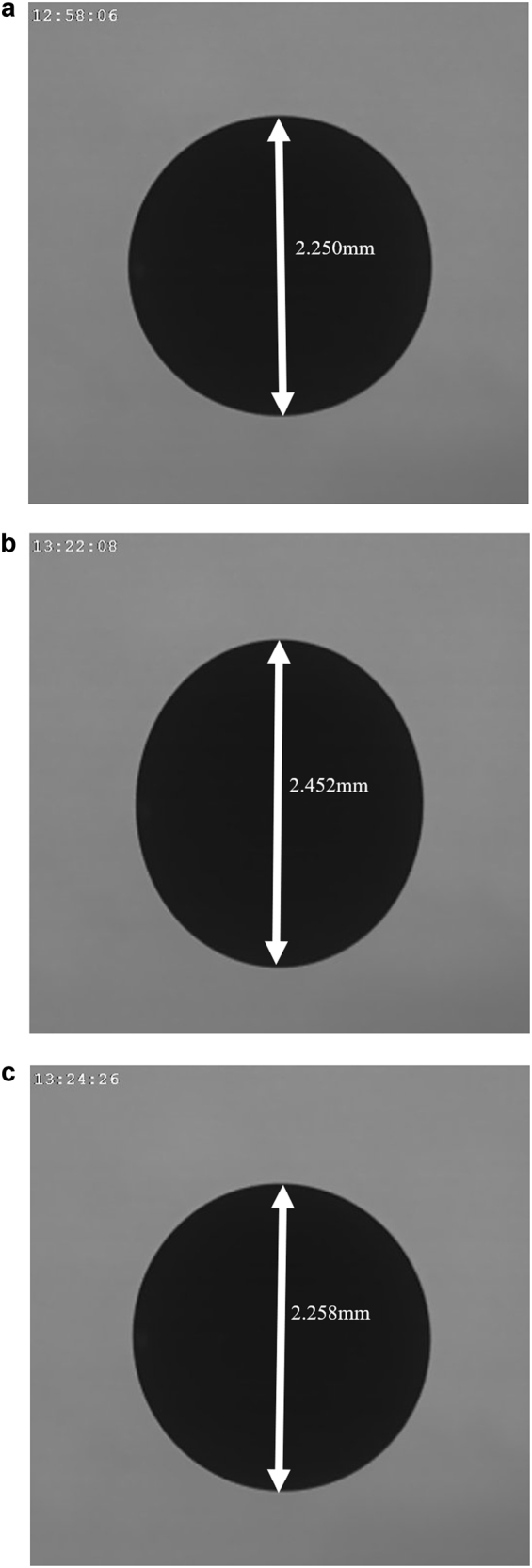
Sample images of levitated Zr sample at 1800 °C during Faraday forcing experiments. The diameter of the sample in the absence of any continuous forcing was 2.222 mm. Maximum disturbance caused through the application of continuous forcing **a** below the resonant frequency (180 Hz), **b** at the calculated resonant frequency (185 Hz), and **c** above the resonant frequency (191 Hz). A video accompanies this study that shows dramatic deviation from the spherical shape at the resonant frequency and the decrease in this deviation both before and after the resonant frequency

**Fig. 2:**
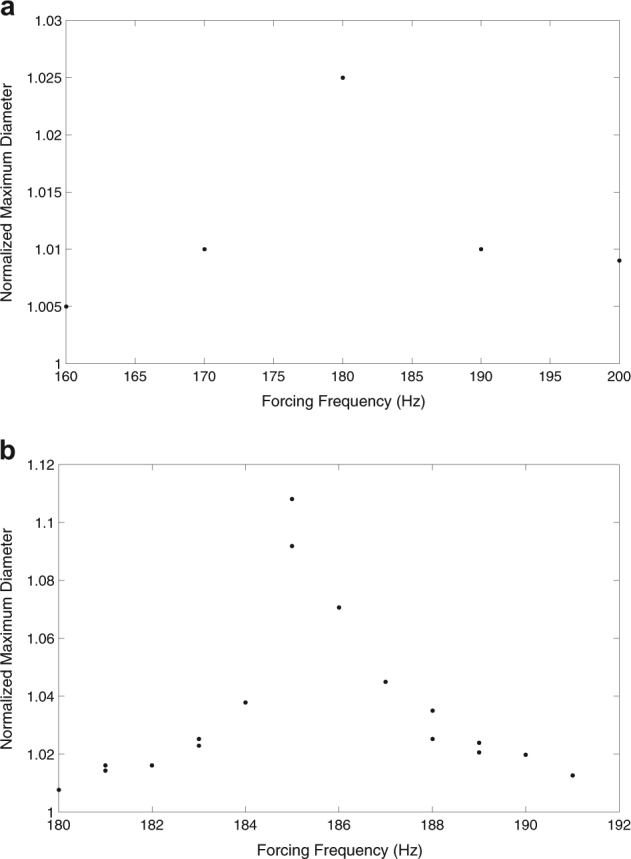
Dependence of the maximum drop diameter upon the forcing frequency. Diameters are normalized by the unforced drop diameter, i.e., the diameter of the sphere. Maximum deviations from the unforced drop diameter are obtained at the resonant frequency. The testing fluid was Zr at 1800 °C, and the amplitude of the imposed oscillation was held constant throughout the tested frequency range. The tested frequency range and frequency step size is initially large, as shown in **a**. After narrowing the frequency range, subsequent experiments are conducted using a smaller step size, as shown in **b**

In each experiment, multiple forcing frequencies are tested at a small, constant amplitude. Images are captured using a high-speed camera and analyzed to quantify the departure of the drop from its spherical shape. Figure 1 describes the change in the deformation of the sample with a slight change in frequency. As can be observed in the figure and in the accompanying video, the magnitude of the maximum departure from the pre-forced spherical shape of a sample is greatly magnified at the resonant frequency. This increase is typically at least 10% greater than the nominal diameter, as seen in Fig. 2.

Table 1 summarizes the surface tension measurements obtained for three samples and companion literature values. Temperatures were chosen in the undercooled region for Zr samples to reduce mass loss via evaporation during the course of experiments, but this was not possible for the Ti_39.5_Zr_39.5_Ni_21_ due to repeated unintentional solidification.

**Table 1:** Table of results for surface tension measurements obtained using the Faraday forcing method

Sample	Temperature (°C)	Mass (mg)	Radius (mm)	Measured surface tension (Nm^−1^)	Literature surface tension (Nm^−1^)	% Difference
Zr	1700	35.101	1.1105	1.47715 ± 0.00783	1.51703^a^^18^	−3%
Zr	1800	35.027	1.111	1.41230 ± 0.00764	1.50603^a^^18^	−6%
Ti_39.5_Zr_39.5_Ni_21_	950	51.439	1.283	1.5320 ± 0.00965	1.670^b^^19^	−14%

Resonant frequencies were found within 0.5 Hz. Surface tension measurements are calculated without the interfacial charge correction shown in Eq. (1), which would slightly increase the values for all experimental runs (~1% increase)

^a ^Error estimated as <5%

^b ^Estimated value based upon Fig. 2 in ref. ^19^ due to discrepancy in provided fit parameters with experimental data. Estimated error in the given fit parameters was reported as ±0.26%

## Discussion

As can be observed, the Faraday forcing method yields surface tension measurements which are typically slightly lower than literature values measured using the current pulse-decay analysis. Approximately 1% of the difference could be recovered by taking the charge correction term shown in Eq. (1) into account during our analysis. We attribute the remaining discrepancy to the fact that the pulse-decay analysis inherently involves the excitation of multiple modes upon delivery of the pulse and subsequent feedback control to maintain the drop in its levitated state. This causes the overall response to be comprised of many modes, with *n* = 2 decaying the slowest. Non-linear interactions between modes will cause the response frequency to shift upward, resulting in higher surface tension measurements after utilization of Eq. (1). To test this hypothesis, decay experiments were also conducted for all three samples.

**Fig. 3:**
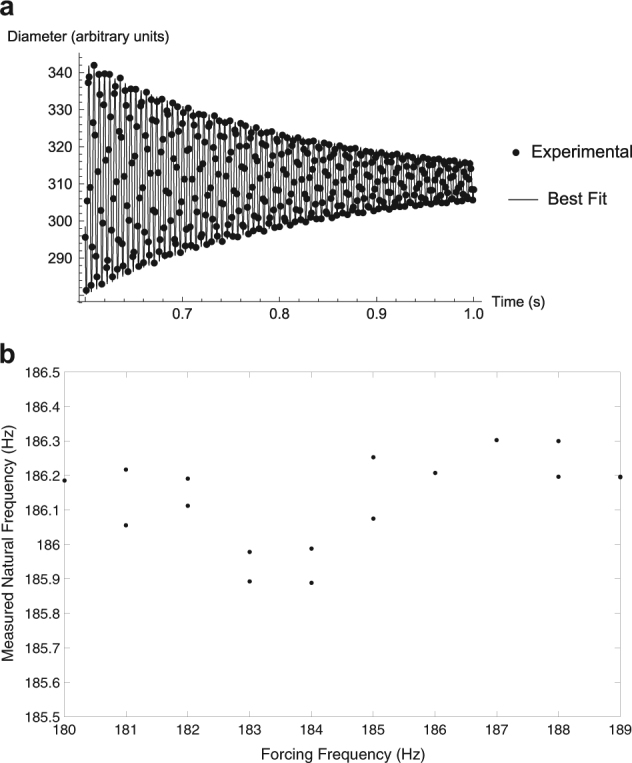
Sample pulse decay analysis data. **a** Sample decay after imposition of an oscillatory voltage at 185 Hz to a Zr sample at 1800 °C. Analysis of the experimental data results in a decay frequency of 186.25 Hz. **b** Dependence of measured natural frequency on the pulsating frequency when utilizing the pulse-decay method. The results obtained using this method are nearly independent of the forcing frequency

Recall from the introduction how the Faraday forcing method distinguishes itself from the pulse-decay method. To further highlight the difference between the methods, the pulse-decay technique was also analyzed for the tested samples. Figure 3a shows a sample of the data and best fit for a Zr sample at 1800 °C pulsed at a frequency of 185 Hz when using the pulse-decay method. By utilizing the pulse-decay technique, the best fit for an imposed pulsating frequency of 185 Hz would yield a natural frequency of 186.25 ± 0.00697 Hz, which is 0.68% higher than that observed using the Faraday forcing technique. The average measured natural frequency for the 1800 °C Zr sample over all of the tested pulsating frequencies was 186.09 Hz. It should be noted that the instantaneous pulsing of the sample at a frequency above or below the resonant frequency determined by the Faraday forcing method did not substantially impact these results, as shown in Fig. 3b. In fact, the decay analysis provided consistent results for the natural frequency regardless of the pulsating frequency. Though this result was consistent, it was always higher than the frequency which produced the maximum magnitude of departure from each sample’s spherical shape, resulting ultimately in discrepancies for the surface tension measurement of between 1.36% and 4.37% for the tested samples. This analysis effectively shows that the two techniques will produce different results for exactly the same sample, though the sample clearly resonates most strongly at the frequency determined by the Faraday forcing method, i.e., the current method.

The larger discrepancy observed between the Faraday forcing result and the literature values for the Ti_39.5_Zr_39.5_Ni_21_ alloy is attributed to possible oxidation of the sample during testing, as these types of alloys are known to be highly sensitive to oxygen contamination.^18^ This was evident through the post-processing analysis of the sample, which showed a mass gain of 0.085 mg. The calculated surface tension matches more closely with sample STL-625 within Bradshaw et al.^19^

Generally, the magnitude of the departure of the drop diameter from its unforced value rises steadily with increasing frequency until the resonant frequency is reached, where a sharp increase is observed. Upon surpassing the resonant frequency, the response decays sharply upon further increase in the assigned frequency. Due to the drastic departures, the resonant frequency range can easily be estimated by conducting a frequency sweep using a large (typically 5 Hz) step size. Subsequent experiments can pinpoint the resonant frequency by taking multiple measurements using a small frequency step size around this estimated range and then conducting image analysis.

Excitations of higher modes were attempted multiple times for all samples. However, the feedback control was not efficient enough to hold the drop in its levitated position when applying the high frequencies and amplitudes required to excite these modes. This resulted in the drop contacting either the top or bottom electrode, thence solidifying. Ideally, higher-order modes would be accessible to provide an additional resonant frequency and resultant surface tension measurement which could be averaged with that obtained from the mode 2 measurement. On ground, the size of the levitated drop is limited due to the voltage requirements necessary for levitation, and also due to small gravitational effects that cause the drop’s natural shape to become more non-spherical with increasing drop size. The use of microgravity would allow for the levitation of larger drops, lowering the natural frequency and thus allowing for the access of higher-order modes.

## Conclusions

In this work, we have described a method for the measurement of surface tension of electrostatically levitated liquid samples at high temperature through the use of continuous Faraday forcing. This forcing offers distinct advantages over the conventional pulse-decay analysis method, particularly when the sample viscosity is high or the levitation feedback control system is noisy. Furthermore, this method involves experimental comparison to an analytical expression without the need for curve fitting. Utilizing this method, fluid samples are continuously oscillated to purposefully excite mode 2 responses over a wide frequency range, and the resultant deformation of the sample is analyzed to determine the resonant frequency, which is taken to be the natural frequency of the drop.

Three surface tension measurements were taken using the Faraday forcing method, and good agreement was observed between these results and those previously published in literature. The Faraday forcing method typically results in surface tension measurements slightly lower than literature values. We believe that these discrepancies stem from non-linear interactions present upon pulse excitation of multiple modes which are induced when using the pulse-decay method due to the feedback control.

Future work should include low-gravity experiments, which could allow for the levitation of larger droplets after modifying existing equipment, lowering the natural frequency and thus allowing for the excitation of higher modes. These higher modes will serve as additional data for confirmation of surface tension measurements. Ideally, the Faraday forcing method should be used in conjunction with the traditional pulse-decay method to allow for accurate determination of both the interfacial tension and the viscosity of a sample.

## Methods

Experiments were conducted using the ESL facility at the NASA Marshall Space Flight Center. Solid samples were initially levitated in vacuum (10^−6^ to 10^−7^ torr) using multiple electrodes coupled to a feedback controller. A UV beam initialized the charge on the sample prior to levitation and replenished the charge throughout the duration of the experiment. A neodymium-doped yttrium aluminum garnet (Nd:YAG) laser was first used to melt the sample, and then density measurements were conducted by raising the temperature until molten and approximately 50 °C superheated before turning off the laser and recording the droplet shape as a function of temperature. A single wavelength pyrometer (LumaSense Technologies IMPAC IGA 140) was used to measure and record the temperature accurately between 300 and 3000 °C. For the determination of surface tension, the temperature was raised above the melting point before allowing the sample to cool to the desired temperature, either superheated, at the melt point, or undercooled, while remaining in the liquid phase. In the case of Zr, experiments were conducted in the undercooled regimes in order to minimize mass loss via evaporation throughout the course of an experiment. This undercooling was not possible for the Ti_39.5_Zr_39.5_Ni_21_ sample due to repeated unintentional homogeneous nucleation. After reaching the desired temperature, a temperature control system maintained the temperature to ensure the sample temperature remained constant during a measurement.

A frequency range is selected by estimating the natural frequency of the material using the measured density and radius of the sample. After the sample is successfully levitated, melted, and cooled to the temperature of interest, an oscillatory forcing is superimposed over the controlled voltage drop at a constant amplitude for all measurements at a given temperature. Each frequency within the expected region is analyzed and the sample response is recorded using a high speed camera. In the experiments, a camera recording at 1000 fps with a resolution of 512 by 512 pixels was used. The resonant frequency is estimated visually for an initially large frequency step size, as shown in Fig. 2a. After this estimation, a narrow range of frequencies around the estimated resonant frequency is selected for further experiments. Within this narrow range, subsequent experiments are conducted until the uncertainty in the determined resonant frequency reaches an acceptable value. Images collected for all frequencies within the narrow range are analyzed using ImageJ® to determine the frequency at which the sample most strongly resonates, i.e., deviates from its spherical shape. The resonant frequency is taken to be the tested frequency which produces the largest prolate and oblate oscillations away from the natural spherical shape of the sample. The smaller the step size, the more accurate the determined resonant frequency. This determined resonant frequency must therefore be the natural frequency of the drop. After obtaining the resonant frequency, Eq. (1) is used to calculate the surface tension of the material for the trial.

### Data availability

All relevant data are available from the authors N.B. and R.N.
